# Volcanic eruptions and public health in Ecuador: an analysis of institutional gaps and integrated strategies

**DOI:** 10.3389/fpubh.2025.1705862

**Published:** 2025-12-17

**Authors:** Katherine Simbaña-Rivera, Damary S. Jaramillo-Aguilar, Jhon Paul Guerrero-Gonzalez, Ruth Jimbo-Sotomayor, Xavier Sánchez, Maurizio Mulas, Luis D. Boada

**Affiliations:** 1 ECUAVOLCAN Research Group, Centro de Investigación para la Salud en América Latina (CISeAL), Pontificia Universidad Católica del Ecuador (PUCE), Quito, Ecuador; 2 Toxicology Unit, Research Institute of Biomedical and Health Sciences (IUIBS), University of Las Palmas de Gran Canaria (ULPGC), Las Palmas de Gran Canaria, Spain; 3 Facultad de Ingeniería en Ciencias de la Tierra, Escuela Superior Politécnica de Litoral (ESPOL), Guayaquil, Ecuador

**Keywords:** risk management, environmental hazards, public health, healthcare systems, volcanic eruptions, Ecuador, one health, risk assessment

## Abstract

Ecuador, a country with high volcanic activity, faces substantial public health risks from frequent eruptions. This review examines the health impacts of recent volcanic events and assesses the national health system’s preparedness and response capacity. Through a critical review of public policies, current regulations, Ministry of Public Health interventions, its inter-institutional coordination and local experiences, this review identifies persistent gaps in governance, operational continuity, primary care, epidemiological surveillance, and risk communication. Both acute and chronic health effects are highlighted, alongside limitations in reaching vulnerable populations. The article proposes an integrated approach grounded in geological, biomedical, and social sciences, framed within the One Health paradigm. Strategic recommendations are presented to strengthen institutional capacity, secure essential resources, and develop a national research agenda on volcanic risk and health. Reducing health impacts require evidence-based policymaking, intersectoral coordination, and sustained community engagement.

## 1 Introduction

Volcanic eruptions generate multifaceted health impacts that extend beyond immediate physical hazards. Acute risks include traumatic injuries, burns, respiratory compromise from ash and gases, and increased susceptibility to water- and food-borne diseases due to disrupted sanitation and contaminated supplies (1–4). Prolonged ash exposure raises concerns for chronic diseases. These effects are often exacerbated in low- and middle-income countries, where health systems face structural constraints such as limited surveillance capacity, shortages of trained personnel, geographic inaccessibility, and fragile infrastructure, conditions that disproportionately affect rural and marginalized populations (5). Volcanic eruptions also disrupt livelihoods, social networks, and access to care (6). This complexity underscores the need for integrated public health strategies.

Ecuador, located in the northwestern region of South America and traversed by the Andes Mountain range, is part of the Pacific Ring of Fire. A total of 84 Quaternary volcanoes have been identified along the Andes (7), of which 27 classified as active or potentially active; about 35% of Ecuador’s population lives in areas at risk from future eruptions (7, 8).

In this geological context, volcanic eruptions pose not only geological hazards but also major public health challenges. Communities located near active volcanoes are at heightened risk due to exposure to volcanic ash and gases (3, 9), which can cause traumatic injuries, respiratory, ocular, dermatological, and mental health impacts including anxiety and stress (10, 11), and contribute to outbreaks of water- and food-borne diseases (12–14). Furthermore, the national health system’s operability is frequently compromised due to infrastructure disruption and surges in healthcare demand—issues exacerbated by uncontrolled urban expansion, access to health services, widespread poverty, low educational attainment, and fragile basic services (15, 16).

The absence of effective mechanisms for inter-agency coordination, disaster management, and risk communication can significantly worsen the outcomes of volcanic crises. In response, Ecuador has prioritized the surveillance of high-risk volcanoes and allocated considerable financial resources to mitigate future risks and protect public health (15, 17, 18). Nevertheless, despite the country’s marked vulnerability, there remains a lack of rigorous evaluations of public health interventions. Evidence regarding their effectiveness, cost-effectiveness, and equity impacts—particularly on vulnerable populations—remains scarce, and their short-, medium-, and long-term outcomes have yet to be systematically assessed (15).

This article aims to analyze Ecuador’s public health preparedness and response to volcanic hazards between 2000 and 2024 by examining health sector policies, interventions, and institutional coordination, aiming to identify gaps and opportunities in risk management, epidemiological surveillance, and emergency response. These insights are intended to strengthen governance, operational continuity, and risk communication, ultimately enhancing the health system’s capacity to manage volcanic disasters. In addition, the study provides actionable recommendations to strengthen health governance and intersectoral coordination, offering an evidence-based framework to improve preparedness and resilience against future volcanic events.

## 2 Methods

This comprehensive documentary review was guided by a central question: how Ecuador has managed volcanic risk and protected public health during the eruptive activity of Reventador, Tungurahua, Cotopaxi, and Sangay between 2000 and 2024. This question structured the inquiry around eruptive activity and hazard characteristics, governance and institutional arrangements, documented health impacts in exposed populations, and the public health policies, interventions, and operational responses implemented during this period.

Document retrieval was carried out using targeted keyword searches (e.g., “volcanic ash”, “health impact”, “risk management”, “Ecuador”). Key variables included eruptive periods and characteristics (e.g., ash columns, pyroclastic flows, lava emissions, and lahars), associated health outcomes (e.g., respiratory, ocular, dermatological, gastrointestinal, and psychosocial effects), and health system responses, including lessons for risk communication and emergency preparedness. Across institutional repositories of the Instituto Geofísico de la Escuela Politécnica Nacional (IG-EPN), the Official Registry of Ecuador, the Ministry of Government, Ministry of Public Health (MPH), and the Servicio Nacional de Gestión de Riesgos y Emergencias (SNGRE) [National Risk and Emergency Management Service]. Additional materials were obtained from the Pan American Health Organization/World Health Organization (PAHO/WHO), major national media outlets, and peer-reviewed literature indexed in MEDLINE/PubMed and Scopus. Searches in institutional and governmental repositories were conducted primarily in Spanish, whereas academic databases were explored in both Spanish and English to maximize retrieval coverage. Data collection was completed between July and August 2025.

Document selection was conducted through independent screening by two reviewers who evaluated titles, summaries, and full texts according to predefined eligibility criteria. Institutional reports, official regulations, scientific publications, and grey literature directly addressing volcanic activity, public health impacts, or emergency responses in Ecuador were eligible for inclusion. Materials were excluded if they lacked a verifiable institutional origin, demonstrated insufficient relevance to volcanic risk and health, or failed to provide minimal contextual or methodological detail when such information was expected.

To ensure analytical coherence, data extraction followed a structured Excel-based matrix, enabling systematic classification of information by source, time period, and thematic category. The analysis was organized into four interrelated dimensions: (i) eruptive history and activity: frequency and duration of eruptive periods, magnitude and type of emissions, and geographic extent of ashfall and affected provinces; (ii) governance and institutional frameworks for disaster risk management within the health sector: legal and regulatory instruments, institutional roles and coordination mechanisms, and risk communication and preparedness plans; (iii) documented health impacts on local populations: reported effects on respiratory, ocular, dermatological, and others; and (iv) public health responses and policy measures: emergency medical response, surveillance and monitoring systems, and community engagement and intersectoral collaboration.

A manual thematic content analysis was applied, combining inductive and deductive coding to identify patterns, relationships, and emergent subthemes across documents. Triangulation of sources and peer verification of coding were implemented to minimize interpretative bias. Discrepancies were discussed until consensus was achieved, strengthening analytical validity and reproducibility.

This study relied exclusively on publicly available secondary data and did not involve human participants; therefore, ethical review was not required.

## 3 Recent volcanic eruptions in Ecuador

Over the past 24 years, four continental volcanoes have erupted. Recent eruptions of Tungurahua (1999–2016), Cotopaxi (2015, 2022–2023), Sangay (1628–present), and Reventador (2002–present) have produced eruptions with substantial regional health, environmental, and socio-economic impacts (14, 16, 17, 19–33) (Table 1).

**Table 1:** Recent major volcanic eruptions in Ecuador: main eruptive characteristics, associated health impacts, and public health responses (20th–21st century).

Volcano	Eruptive period (years)	Main characteristics	Health impacts	Health response/lessons learned
Reventador	2002 – present. Previous intermittent activity in 1898–1912, 1926–1929, 1944, 1959–1960, 1972–1974, and 1976.	Sudden eruption northeast of Quito in 2002 with ash plumes, pyroclastic flows, lava, and lahars; nearly continuous activity since then (20).	Respiratory, ophthalmological, gastrointestinal, and dermatological conditions; contamination of potable water sources.	Public health emergency due to lack of safe water; highlighted vulnerability of water and sanitation infrastructure (14).
Tungurahua	1999–2016. Previous reactivation in 1916–1918. Major eruptive crises in 2006 and 2010–2014 (21, 22).	Prolonged eruptive phase with Strombolian, Vulcanian, and pyroclastic events; recurrent evacuations of Baños and agricultural areas (23).	Respiratory and ocular irritation; agricultural losses with nutritional repercussions; psychosocial issues due to evacuations (24).	Consolidation of the community-based *“vigías del Tungurahua”* model as a benchmark for early warning and risk communication (25).
Cotopaxi	2015 and 2022–2023. Historical activity in 1903–1904, minor eruptions until 1940; unrest signals in 1975–1976 and 2001–2002 (26).	Reactivation after >70 years of dormancy; phreatic and phreatomagmatic eruptions; ash columns up to 2 km; secondary lahars (27).	Risk to over 300,000 inhabitants; respiratory and ocular conditions; potential large-scale impact from lahars.	Activation of MPH contingency plans; preventive campaigns on mask use, eye protection, and safe water (28).
Sangay	2019–present. Near-continuous activity since 1934, with intense phases in 1934–1937, 1941–1942, 2001–2013, and 2015–2018 (29).	High-altitude ash plumes dispersed hundreds of kilometres; lava emissions (172 ± 86 million m^3^ in 2020) (30).	Respiratory and ocular conditions; risk of fluorosis from fluoride-rich ash; contamination of water and soils (19).	Public health alerts and educational campaigns; preventive measures (mask use, safe water storage).

These events disrupted essential services, affected air and water quality, and generated acute respiratory, ocular, dermatological, and gastrointestinal illnesses among exposed populations (14, 19, 24). Yet, no systematic epidemiological surveillance has assessed medium-term consequences, such as potential increases in chronic respiratory disease or other reported conditions, revealing a persistent gap in post-eruption health monitoring. While the “*vigías*” initiative at Tungurahua exemplifies the value of community participation in early warning (25), integration of the health sector into these grassroots networks remained minimal. Likewise, public health campaigns have not been systematically assessed for their effectiveness in reaching rural and Indigenous populations, and the lack of biomonitoring of chronically exposed communities has hindered understanding of potential long-term outcomes.

## 4 Governance of disaster and risk management in the health sector

Disaster and risk management governance in Ecuador is principally organized under the SNGRE (34). A key technical actor in this system is the IG-EPN, responsible for nationwide seismic and volcanic monitoring, facilitating timely and reliable early warning systems (35). However, while technical monitoring is robust, the integration of this information into public health decision-making remains limited, with alerts often prioritized for civil protection rather than health system preparedness.

The Organic Health Law, enacted in 2006, underscores the integration of risk management into emergency and disaster preparedness. It mandates the establishment of comprehensive information systems, continuous staff training, and emergency plans in all public and private healthcare facilities to enhance prevention and response capacity (36). Yet, evaluations of compliance with these mandates remain scarce, and it is unclear to what extent such provisions have been consistently operationalized across different regions and levels of the health system. In parallel, the 2008 Constitution of the Republic of Ecuador, recognizes disaster risk management as essential to the well-being of the population, thus assigning explicit responsibilities to the state (37).

In alignment with these legal mandates, the MPH has established the Risk and Damage Management Directorate (38). This office is tasked with planning and implementing measures for prevention, preparedness, mitigation, response, and epidemiological surveillance related to natural or anthropogenic hazards affecting the health sector, ensuring compliance with national regulations. However, the Directorate’s limited technical staff and resources restrict its ability to fully coordinate with decentralized health districts, particularly in high-risk volcanic areas.

The 2024 Organic Law on Comprehensive Disaster Risk Management reaffirmed SNGRE’s authority over national policy in this domain (34). According to this law, the MPH is designated as a core agency in humanitarian response, mandated to effectively mobilize its logistical capabilities and ensure the immediate availability of resources during disasters. Nevertheless, the degree to which these responsibilities translate into effective preparedness for vulnerable populations, including indigenous communities, people with disabilities, and those with chronic diseases, remains uncertain.

## 5 Health impacts of volcanic eruptions

Volcanic eruptions exert a wide range of adverse effects on human health, both direct and indirect. In the acute phase, pyroclastic flows, sudden explosions, and ballistic projectiles can result in severe trauma, extensive burns, inhalational asphyxiation, and in extreme cases, death (39). One of the most widespread hazards is volcanic ash—composed of fine vitric particles and gaseous compounds, particularly sulphur dioxide, hydrogen sulfide, hydrochloric acid, hydrofluoric acid—which, when inhaled, is associated with acute respiratory effects and exacerbation of pre-existing conditions such as asthma and bronchitis. Ash exposure may also cause pharyngeal irritation, persistent dry cough, conjunctivitis, corneal abrasions, and contact dermatitis (9, 40). Acute exposure to hot ash can lead to burns of the respiratory mucosa, while chronic exposure to ash containing crystalline silica, fluoride, and heavy metals has been linked to long-term illnesses, including skeletal fluorosis, silicosis, and a potentially elevated risk of thyroid and pulmonary cancers, according to emerging evidence (1, 41).

Moreover, the deposition of ash on surface water sources and agricultural land compromises drinking water quality and food safety, increasing the risk of gastrointestinal illnesses and waterborne diseases (4). However, health system contingency plans rarely incorporate explicit water, sanitation, and food safety monitoring protocols, despite repeated evidence of contamination events. The accumulation of ash may also cause the collapse of roofs and fragile structures, posing additional trauma risks and prompting population displacement (39). Indirect health consequences include the deterioration of food security due to the death of livestock, crop failure, and soil contamination, further exacerbating nutritional vulnerability in affected communities (4). These cascading effects often translate into catastrophic economic losses that restrict access to essential services, including health care, and are particularly severe in rural communities reliant on subsistence agriculture, where food insecurity is already prevalent.

Beyond the physical health burden, volcanic eruptions generate significant psychosocial stress, manifested as anxiety, distress, sleep disturbances, and post-traumatic stress disorder (PTSD) (2). However, these effects fluctuate over time and are influenced by exposure level, duration of eruptive activity, social support, and individual resilience (6). Studies conducted after the eruptions of Eyjafjallajökull (Iceland), Mt. Merapi (Indonesia), and Mt. Miyakejima (Japan) documented persistent anxiety, depressive symptoms, and PTSD among exposed populations, particularly in those facing recurrent ashfall or displacement (42–46). Nonetheless, as highlighted by López-Vázquez and Marván (47), residents of volcanic areas often develop active coping mechanisms that sustain functionality despite chronic stress exposure. Such variability underscores that mental health outcomes are not solely determined by proximity to volcanic hazards but by the interplay of environmental, social, and psychological factors, emphasizing the need to incorporate psychosocial care into volcanic risk management and emergency response strategies.

In addition, volcanic activity often disrupts basic services and the functionality of the health system. Dense ash reduces visibility, impeding ground and air transport, which delays evacuation from high-risk areas and the timely transfer of patients. Ash infiltration into water capture, distribution, and sewage systems can obstruct entire networks, interrupting access to safe water and generating secondary health risks (14). Critical infrastructure, including roads, water treatment facilities, and electrical networks, may be damaged by ash accumulation or lahar flows, isolating entire communities and limiting institutional response capacity. Healthcare facilities in volcanic zones may become non-operational due to structural damage, power outages, water supply issues, or precautionary staff evacuations (39). Given these vulnerabilities, volcanic risk management must ensure the operational continuity of health services. Yet, few hospitals in Ecuador have been independently evaluated against international safe hospital standards, and drills simulating volcanic emergencies remain limited in scope (48). This includes protecting infrastructure, implementing contingency plans, and guaranteeing the provision of medical care even under prolonged emergency conditions.

## 6 Public policy and health planning in volcanic risk management in Ecuador

### 6.1 Role of the Ministry of Public Health in volcanic risk governance

In response to volcanic eruptions over the past two decades, the MPH through National Directorate of Risk and Damage Management has implemented various strategies in coordination with the SNGRE and other governmental entities (34, 36, 37). This intersectoral collaboration has enabled the development and execution of measures aimed at disease prevention, health promotion and hygiene in emergency contexts, the continuity of comprehensive medical care—including mental health services—epidemiological surveillance in exposed populations, and sustained provision of safe water and basic sanitation. Partnerships with international organizations have also been consolidated to facilitate technical cooperation, humanitarian assistance, and timely access to essential supplies and specialized equipment (49, 50). Nevertheless, most of these actions remain reactive and short-term, with limited evidence of institutionalization into routine public health planning or integration into local health district strategies.

Hazard-specific contingency plans, developed by SNGRE for each volcano, were disseminated as part of the national preparedness strategy. However, these plans were not revised or updated following the 2015 Cotopaxi eruption until mid-2023. The resumption of this process led to substantial improvements, particularly concerning health system preparedness and inter-institutional coordination (51). In this context, the MPH has led the planning and execution of both local and national simulation exercises to validate response protocols, assess operational readiness, and promote civic co-responsibility in volcanic emergencies (52–54). Yet, evaluations of these simulations have rarely been published, and lessons learned are not systematically incorporated into subsequent planning cycles, reducing opportunities for continuous improvement.

### 6.2 Targeted interventions led by the Ministry of Public Health

MPH-led initiatives have remained relatively limited and have mostly occurred following the 2015 Cotopaxi eruption. Despite the existence of the Risk and Damage Management Directorate within the MPH, the SNGRE has highlighted significant shortcomings in risk management and impact assessment (54). This reactive pattern illustrates the absence of a sustained prevention culture within the health sector, with preparedness activities often depending on the immediacy of eruptive crises rather than long-term planning.

In response, the MPH allocated dedicated budgetary resources in 2016 to launch the “Contingency Project to Prevent the Effects of the *El Niño* Phenomenon, the Potential Eruption of Cotopaxi Volcano, and Other Natural Hazards.” Its primary objective was to ensure uninterrupted health services across 120 vulnerable health districts (52, 55). Key components included strengthening the equipment of ambulances, mobile hospitals, and air support for emergency care; enhancing vector control and occupational safety capacities; and training healthcare personnel in advanced life support, trauma management, and specialized pediatric care (55). However, no independent evaluation of this project’s effectiveness, coverage, or sustainability has been reported, making it difficult to determine its real contribution to long-term health system resilience.

Since 2021, the MPH has developed susceptibility maps for health facilities exposed to volcanic threats (including Cotopaxi, Tungurahua, Reventador, Sangay, Cayambe, Chiles, and Cerro Negro), as well as other natural hazards (51). The MPH has also produced post-event situation reports and, through its Directorate of Epidemiological Surveillance, actively monitored acute health effects linked to recent eruptions, deploying technical and human resources according to the scale of impact (56). Although, these maps and reports remain largely internal documents, with limited public accessibility, which reduces transparency and hinders opportunities for academic validation and community engagement.

Operationally, health districts and facilities are now required to develop annual multi-hazard response plans aligned with the PAHO/WHO Health Sector Response Framework (57). These plans include the establishment of risk management committees, technical training of staff, execution of drills and simulations, and evaluation of evacuation routes, emergency exits, and safety zones within healthcare infrastructure (34, 52, 53).

To enhance communication with the public, the MPH has trained focal points in community health risk education and used official communication channels to disseminate key guidance during volcanic events, including biosafety and self-care recommendations (58).

## 7 Gaps and challenges in health sector response

Despite advances in preparedness, critical gaps remain in the Ecuadorian health sector’s response to volcanic emergencies. These are summarized as follows:

### 7.1 Institutional governance and risk management

Most public sector institutions, including those within the health domain, either lack formally established Risk Management Units (RMUs) or maintain units that are under-resourced and lack clear mandates. Risk management is not consistently integrated across institutional planning processes (54), undermining the capacity for anticipation, coordination, and effective response (53, 59). The absence of dedicated budget lines further weakens institutional commitment, resulting in dependence on ad-hoc funding during crises.

### 7.2 Communication and cultural adaptation

Preventive messages and alerts have historically been disseminated primarily in Spanish, creating a structural barrier in Ecuador’s multicultural context. Approximately 4.5 million Ecuadorians predominantly speak *Kichwa* or other Indigenous languages, yet multilingual educational materials and culturally adapted formats (oral, visual, radio-based) remain largely unavailable for Indigenous and rural populations with low literacy levels (60). However, communication gaps extend beyond linguistic translation. They reveal deeper misalignments with sociocultural dynamics and worldviews that shape risk interpretation, collective decision-making, and trust in institutions. Indigenous and *campesino* communities operate within living cultural systems in which oral transmission, community assemblies, symbolic meaning, and experiential knowledge play central roles. The failure to integrate these dimensions, coupled with the reliance on Spanish-language and written materials, has weakened institutional credibility, reduced message uptake, and contributed to delayed evacuations or reluctance to adopt protective health measures.

### 7.3 Training and local resource limitations

Health posts in high-risk rural areas often lack both specific training and the necessary supplies to manage volcanic-related conditions, such as treatment of burns or acute respiratory distress due to ash exposure. Moreover, staff turnover in rural posts undermines the retention of trained personnel, perpetuating vulnerability. The absence of pre-positioned Personal Protective Equipment (PPE) (e.g., N95 masks, protective goggles, water filters) was evident during past eruptive events, although mask availability has improved since the COVID-19 pandemic (61). Many of the tools and protective devices distributed during emergencies are designed for urban or industrial settings rather than for agricultural and field-based work, and community members frequently report usability problems. Masks become damp during physical labor, goggles fog in humid conditions, and heavy boots or impermeable suits restrict movement on steep or muddy terrain. Low uptake therefore reflects practical barriers rather than refusal. These challenges coexist with significant and recurrent resource gaps, particularly in ambulance coverage, cold chain maintenance of essential medicines, and stockpiles for respiratory conditions and chronic disease management during prolonged crises (62).

### 7.4 Integration of health into risk planning

Despite the existence of SNGRE, coordination between technical (e.g., volcanology) and health sectors remains weak. Long-term health impacts of volcanic activity have not been systematically assessed. For instance, following the 2002 eruptions, authorities informally reported “no persistent effects,” yet no systematic epidemiological surveillance was conducted to confirm this (60). This lack of data impedes the identification of post-eruption health needs; such as increases in chronic respiratory diseases or mental health disorders. This absence of surveillance not only impedes identification of post-eruption needs but also prevents the health system from generating evidence to inform preventive investment and long-term rehabilitation programs.

### 7.5 Operational continuity of health services

A critical challenge lies in ensuring that hospitals and health centers in hazard zones possess robust contingency plans that are regularly tested, updated, and aligned with PAHO’s Safe Hospital standards (48). Essential facilities may lack resilient infrastructure (e.g., alternative water or power systems, patient evacuation protocols) necessary for major eruptions. Past experiences show that basic service disruption severely compromises hospital function (39). There are persistent and widespread deficits in simulation exercises involving eruption scenarios, including validation of hospital evacuation routes, transport of chronically ill patients, emergency supply chains, referral systems for specialized care, and cross-provincial coordination.

### 7.6 Human resources and structural financing

The health system’s response capacity is severely constrained by chronic staff turnover, absence of incentives to retain trained personnel in rural areas and limited structural funding for preventive actions. These challenges are compounded by the absence of consolidated public health teams with clearly defined technical profiles, merit-based recruitment, and continuous training programs. This results in a reliance on temporary staff and volunteers during crises, which compromises the continuity and quality of health services. Additionally, Decentralized Autonomous Governments (DAGs) and health institutions lack dedicated and mandatory budget lines for disaster risk reduction activities (48).

### 7.7 Intersectoral and institutional coordination

The articulation between state institutions, the health system, academia, and international cooperation agencies is inconsistent and often dependent on personal networks rather than institutionalized mechanisms. Actions are often fragmented and reactive, rather than coordinated through proactive intersectoral planning. Technical inter-institutional committees focused on health and disaster risk are largely absent, as are local coordination networks involving key stakeholders (60). This hampers knowledge translation from academic research into policy and reduces opportunities to align international aid with national priorities.

### 7.8 Primary care coverage and specialized surveillance

During previous volcanic emergencies, healthcare services prioritized urgent cases (e.g., trauma, acute respiratory infections), while other areas received inadequate attention often neglecting preventive and follow-up care. These included mental health services, outbreak control (e.g., diarrheal diseases from contaminated water), and continuity of care for chronic patients and continuity of care for chronic patients, including those requiring dialysis, insulin therapy, tuberculosis treatment, or antiretroviral treatment. These gaps were compounded by the absence of systematic post-event monitoring, as seen after eruptions such as Tungurahua, which has limited understanding of long-term physical and mental health impacts. Testimonies from the *vigías* and affected communities describe persistent changes in wellbeing, behavior, and social functioning that remain undocumented and untreated. As expressed in the *Luna de Maíz* Podcast (63), several *vigías* recount that “life did not return to what it was before,” noting ongoing fear, disrupted routines, and the feeling that “when the volcano quieted, the institutions quieted too.” Despite their critical role in risk monitoring, these groups often report institutional abandonment once eruptive activity declines. Together, these shortcomings underscore the need for comprehensive health protocols that address both physical and mental health in disaster contexts, ensure the continuity of services, and strengthen both routine and syndromic epidemiological surveillance systems capable of capturing medium- and long-term outcomes, including uncommon post-eruption health effects (e.g., fluoride toxicity, stress-induced preterm births).

## 8 Actionable recommendations for strengthening health governance and intersectoral coordination

To enhance volcanic risk management from a public health perspective, several strategic policy and action-oriented recommendations are proposed:

### 8.1 Institutionalizing health risk governance

Strengthening disaster risk governance in the health sector requires the formal establishment of RMUs at all institutional levels—central, zonal/district, and healthcare facilities—with clearly defined mandates, technical profiles, and permanent budgets. Equally important is ensuring that RMUs report on performance indicators, such as preparedness drills completed, health facilities evaluated, and proportion of staff trained. These RMUs must be systematically integrated into Emergency Operations Committees and local risk reduction structures (54). Binding regulations should require all public institutions, including health services, to implement RMUs that include technical frameworks, mechanisms for community participation, evaluation systems, and locally adapted ordinances. Legal reforms must guarantee not only the creation but also the financing and accountability of DAGs-level units, to prevent them from existing only on paper.

To ensure effective public health responses to volcanic activity, interinstitutional coordination among the IG-EPN, the MPH, and SNGRE must be significantly enhanced. Volcanic alerts—based on seismic tremor patterns, gas emissions, and ash plume forecasts—must be translated into timely and actionable health interventions. Due to the technical complexity of IG-EPN communications, SNGRE should act as a liaison, converting scientific alerts into operational protocols adapted to the health sector. The lack of standardized frameworks, such as internationally recognized color-coded volcanic alert systems, hinders effective communication and coordination. Adopting such frameworks would improve consistency and facilitate cross-sector understanding of hazard severity and appropriate actions (39). Additionally, emergency response committees should formally include public health professionals to ensure that shelters are adequately protected from ash exposure and that the needs of vulnerable populations—such as individuals with disabilities, children, older adults, and pregnant women—are addressed in preparedness and response plans.

### 8.2 Enhancing local health system preparedness

Improving the resilience of primary healthcare systems in high-risk volcanic zones is essential for an effective emergency response. Healthcare personnel must be trained in managing clinical emergencies with a focus on primary care providers, nurses, and community health workers, who are often the first point of contact in rural zones, specific to volcanic activity, including burns, trauma from pyroclastic flows, mass respiratory distress, and epidemiological control in shelters. Facilities should be equipped with PPE, emergency kits, and standardized, context-adapted clinical protocols, integrated into national guidelines. Health centers must develop contingency plans that address alternative water and energy supplies, protection of water sources from ashfall, and secure evacuation routes for hospitalized patients (39).

Regular multi-stakeholder simulation exercises should assess response capacity and promote coordination among health personnel, local authorities, and communities. These drills must be incorporated into territorial planning and comply with safe hospital criteria, particularly in provinces with high volcanic threat such as Morona Santiago, Tungurahua, and Cotopaxi (48).

### 8.3 Strategic logistics for health emergency response

Implementing a supply prepositioning strategy in high-risk zones is critical for timely and effective health responses. Upon elevation of volcanic alert levels (yellow or orange), district health authorities should activate readiness protocols, including reinforcing regional stocks of high-efficiency masks (N95 or above), protective eyewear, bronchodilators, ophthalmic solutions, dressings, and safe water kits (filters, purification tablets) as well as supplies for continuity of care for chronic diseases (insulin, dialysis consumables, antibiotics, antiretrovirals). These resources must be deployed efficiently to isolated communities affected by lahars, ashfall, or road blockages, with equity criteria guiding resource allocation.

Standardized clinical protocols should be developed for treating acute respiratory conditions, asthma, chronic obstructive pulmonary disease, ocular and dermatological injuries, and burns. Rapid response mobile brigades, specifically trained for volcanic emergencies and equipped with 4×4 vehicles and all-terrain ambulances, must be deployed efficiently to isolated communities affected by lahars, ashfall, or road blockages.

### 8.4 Water, sanitation, and food safety

Intersectoral protocols must be established to safeguard potable water sources and sanitation systems during volcanic events (14). Measures include closing surface water intakes at the onset of ashfall, deploying emergency filtration systems, and providing additional chlorination or bottled water to affected communities. At the agricultural level, response frameworks should assume the possibility of significant or even total crop and forage loss, even under low ashfall scenarios, requiring contingency plans that integrate both technical guidance and traditional food preservation practices, such as the storage of dry grains and seeds, which have historically supported community resilience during ash-induced crises. Training for farmers and livestock producers is critical to prevent contamination, minimize economic losses, and protect nutritional health. These activities should be co-developed with agricultural extension services, ancestral knowledge holders, and public health teams under a One Health approach, ensuring that agricultural safety guidelines and preparedness actions are culturally relevant, feasible, and aligned with human, animal, and environmental health needs.

### 8.5 Risk communication and community health education

Permanent health education campaigns on volcanic risks are essential for fostering community resilience, but current strategies have struggled to incorporate community worldviews, experiential knowledge, and diverse cultural practices. Efforts must move beyond distributing printed materials and instead actively engage community leaders, teachers, health workers, and volcano *vigías* in culturally and technically appropriate message dissemination (60). While multilingual translation has improved, many advisories remain misaligned with local interpretations of volcanic processes, collective organization, and ancestral coping strategies. Communication must therefore be adapted not only linguistically but also to the cosmovision, symbolic frameworks, and lived experience of Indigenous and rural communities that have coexisted with active volcanoes for generations. Diversifying formats, including community radio, visual infographics, and oral presentations in communal assemblies, ensures access for low-literacy populations. Key content should include correct mask usage, safe water hygiene practices, preparation of household emergency kits with essential medications, and early recognition of warning signs. Training local health promoters as information multipliers and integrating disaster preparedness into rural school curricula can further strengthen a preventive culture. Clear, timely, and culturally respectful communication builds public trust, supports effective evacuations, and enhances compliance with protective health measures.

### 8.6 Integrated epidemiological surveillance and post-disaster care

Syndromic surveillance systems should be implemented and linked with existing national health information systems to avoid parallel data silos during and after eruptions to rapidly detect outbreaks or emerging health issues such as acute respiratory infections, diarrheal diseases, injuries, eye irritation, and mental health disorders. Data must be shared in real-time with the SNGRE to guide operational decisions—for example, deploying eye drops to shelters reporting conjunctivitis cases or increasing water supplies during gastrointestinal outbreaks (64). Equity-sensitive indicators (e.g., disaggregated by sex, age, ethnicity, and disability status) are essential to identify disproportionate impacts.

Evidence indicates that ashfall significantly increases healthcare demand for respiratory and ocular conditions (9, 40). Active surveillance enables timely mobilization of specialized medical teams and medications. Mental health and rehabilitation services should be systematically integrated into emergency response policies through the deployment of trained psychologists and social workers to provide psychosocial support and identify post-traumatic stress disorders.

Ensuring treatment continuity for patients with chronic conditions is critical during and after emergencies. Additionally, epidemiological monitoring systems must be established to identify medium- and long-term sequelae.

### 8.7 Research and continuous improvement

Establishing a national research agenda on public health and volcanic risk is vital to generate scientific evidence that informs effective preparedness and response policies. Research priorities should include chronic exposure to volcanic ash and toxic compounds, short- and long-term health effects, biomonitoring of contaminants, and evaluation of the effectiveness and cost-effectiveness of health interventions, including mental health and psychosocial support strategies, which are often neglected. A national data repository and mandatory post-event technical reporting system should be developed in alignment with the Sustainable Development Goals outlined in Ecuador’s 2030 Agenda (65).

Despite isolated advances, such as environmental studies following the 2010 Tungurahua eruption (23), significant gaps remain in evaluating long-term population health indicators. Prolonged exposure to heavy metals or potential endocrine disruptors in high-risk areas warrants further investigation into their association with chronic and oncological diseases, which have not yet been systematically studied in Ecuador. Strengthening environmental health surveillance and developing an inter-institutional, academically supported database is essential to inform policy and regulation. Every simulation and real emergency should conclude with evaluative reports to support continuous improvement in risk management.

### 8.8 Interdisciplinary and holistic approaches

Volcanic risk management and public health demand interdisciplinary collaboration across natural, social, and biomedical sciences. Studies in Ecuador and globally have shown that understanding geological threats alone is insufficient; it is equally important to analyze social dynamics, health determinants, and community risk perceptions. This requires institutional mechanisms that bring volcanologists, epidemiologists, social scientists, and local leaders into routine planning, not only crisis response.

Integrated technical teams composed of volcanologists, epidemiologists, sociologists, healthcare professionals, and communicators should coordinate actions throughout the preparedness, response, and recovery phases. The One Health approach is especially pertinent in eruptive scenarios, facilitating synergistic responses to challenges at the human-animal-environment interface, such as water contamination, impacts on livestock production, and emergence of zoonotic or environmentally linked diseases.

Living with active volcanoes requires a comprehensive strategy combining geophysical monitoring technologies, early warning systems, public health preparedness, participatory research, and community education. While eruptions are inevitable natural phenomena, their escalation into disasters depends on effective anticipation, governance, and evidence-based public policies.

## 9 Discussion

This review delineates persistent structural challenges and emerging opportunities in Ecuador’s public health response to volcanic hazards. Over the past two decades, the MPH has implemented intersectoral strategies aimed at mitigating the health impacts of volcanic eruptions, encompassing disease prevention, epidemiological surveillance, continuity of essential medical services, and the provision of safe water and sanitation (51, 52, 55, 56). Technical cooperation with international agencies have supported capacity-building, humanitarian assistance, and access to specialized equipment (34, 52, 53, 58). Despite these advances, most interventions remain predominantly reactive, episodic, and insufficiently institutionalized within routine governance structures, reflecting longstanding deficits in a prevention-oriented planning and culturally adapted communication (53, 54, 59). Rural posts continue to lack adequate training, equipment suitable for agricultural setting, and mechanisms to incorporate community knowledge.

Comparative analysis with international volcanic crises reinforces these systemic limitations. In Colombia (e.g., Nevado del Ruiz) and Guatemala (e.g., Fuego), insufficient preparedness, limited surge capacity, and weak community engagement reduced evacuation compliance and magnified health impacts (66, 67), mirroring operational gaps seen in Ecuador (39, 60–62). Experiences in Indonesia (e.g., Mt. Agung, Mt. Merapi, Mt. Sinabung) and the Philippines (e.g., Pinatubo) highlight how miscommunication, often rooted in divergences between institutional messaging and local worldviews, contributed to delayed protective actions and erosion of trust (68, 69). These dynamics parallel Ecuador’s context, where risk messages frequently omit Indigenous cosmovision, oral traditions, and local decision-making systems central to rural Andean communities.

Long-term mental health and psychosocial consequences are another recurring theme globally. Studies from volcanos in Iceland, Indonesia, and Japan documented sustained anxiety, somatic symptoms, and behavioral changes, particularly where communities perceived institutional neglect (42–46). Similar challenges emerged in Montserrat (e.g., Soufrière Hills), where chronic displacement disrupted social cohesion and continuity of care for chronic diseases (70). Such findings resonate with reports from Ecuadorian *vigías* who described psychological distress during prolonged eruptive periods—impacts that remain largely unmonitored by the MPH. Conversely, volcanic Small Island Developing States (SIDS) (e.g., Samoa, Tonga, Vanuatu) illustrate how culturally grounded psychosocial support and community-driven recovery efforts can strengthen resilience and accelerate normalization (71).

Operational constraints identified in Ecuador also align with global patterns. Although ashfall-related public health impacts have been widely documented in countries affected by recurrent eruptions, fully integrated environmental-clinical surveillance systems remain scarce worldwide (2). Instead, most evidence comes from temporary monitoring initiatives or research-driven collaborations implemented during or after major eruptions, such as those conducted in United Kingdom as a result of the Icelandic volcanic ash plume in 2010 (72). These experiences nevertheless illustrate the value of linking air-quality measurements, ash characterization, and clinical registries to guide immediate protective actions and refine public health recommendations. Ecuador lacks comparable long-term monitoring frameworks, limiting the ability to track chronic health burdens or evaluate exposure-specific risks. By contrast, the Cumbre Vieja (Spain) response demonstrated strengths in anticipatory governance, particularly in communication, logistics, and continuity of essential services, from which Ecuador could derive important lessons for future system strengthening (73).

Across contexts, resilience improves when authorities collaborate with community leaders, traditional knowledge holders, and local volunteer networks (74). Evidence from SIDS, Democratic Republic of Congo (e.g., Virunga Volcanic Province), and Indonesia (e.g., Mt. Merapi, Mt. Kelut) shows that integrating indigenous environmental indicators, community-led early warning systems, and traditional food storage practices enhances preparedness, improves adherence to protective measures, and facilitates recovery, particularly in agrarian settings where ash threatens food security even at low deposition levels (71, 74–79). Ecuador’s extensive experiential knowledge, especially among volcano *vigías*, remains underutilized within formal protocols (39, 53, 59, 61, 62).

These experiences further emphasize the criticality of standardized epidemiological surveillance, stratified data collection, and sustained community engagement (66, 67, 73, 79). Ecuador currently lacks longitudinal monitoring frameworks, limiting the capacity to generate robust evidence for targeted interventions and policy formulation. Nevertheless, Ecuador holds the potential to integrate geospatial, environmental, and health datasets within a One Health framework, enabling predictive risk modeling and proactive decision-making (39). Institutionalizing RMUs, formalize intersectoral committees, conduct regular simulation exercises, preposition medical and protective supplies, and develop standardized clinical protocols for exposure-specific conditions are essential steps (14, 39, 54). Embedding culturally adapted risk communication into routine operations, linking health monitoring with geological and socio-environmental data, and extending both the duration and cultural training of response teams are critical to enhancing system resilience, reducing dependence on external assistance, and improving intervention efficacy (60). Research priorities should focus on long-term epidemiological surveillance, rigorous evaluation of public health interventions, and identification of population-level vulnerabilities, with particular attention to marginalized and high-risk communities (23).

### 9.1 Strengths and limitations

This study delineates both the strengths and limitations of Ecuador’s public health framework for managing volcanic hazards. It constitutes an initial critical appraisal intended to inform the optimization of risk and disaster management processes within the MPH, emphasizing governance structures, administrative coordination, and operational capacities. Given the absence of peer-reviewed scientific literature specific to Ecuador, the analysis integrates empirical observations from domestic volcanic events alongside international case studies from regions with comparable volcanic risk profiles. Notably, considerations pertaining to hospital infrastructure and primary care delivery were outside the scope of this work, but their inclusion in future research could substantially enhance health system resilience.

## 10 Conclusion

Volcanic eruptions in Ecuador pose a complex threat to public health, with impacts ranging from acute medical emergencies to underestimated chronic health effects. Although regulatory and operational progress has been made, significant gaps persist in health preparedness, continuity of essential services, and specialized epidemiological surveillance, and the integration of health equity considerations into policy and practice. Infrastructure in at-risk areas often lacks the resilience to withstand prolonged emergencies, particularly in rural and marginalized communities, underscoring the urgency of targeted investments in safe hospitals and resilient primary care networks.

Equally urgent is the strengthening of governance in health risk management, institutionalizing technical units across all health system levels, strengthening local operational capacity, and guaranteeing equitable access to strategic resources before, during, and after eruptive events. These measures must be accompanied by mechanisms that systematically incorporate community perspectives, clarify roles in health protection, and identify barriers to cooperation, particularly in settings where trust in institutions and continuity of care have historically been fragile. A national research agenda on health and volcanism is also essential, encompassing environmental surveillance, long-term health outcomes, mental health, and systematic evaluation of interventions, to inform evidence-based policymaking.

Living alongside active volcanoes requires sustained evidence-based policies, robust intersectoral coordination, resilient health systems, and genuine and effective community engagement to minimize risk, reduce inequities, and enhance resilience.
